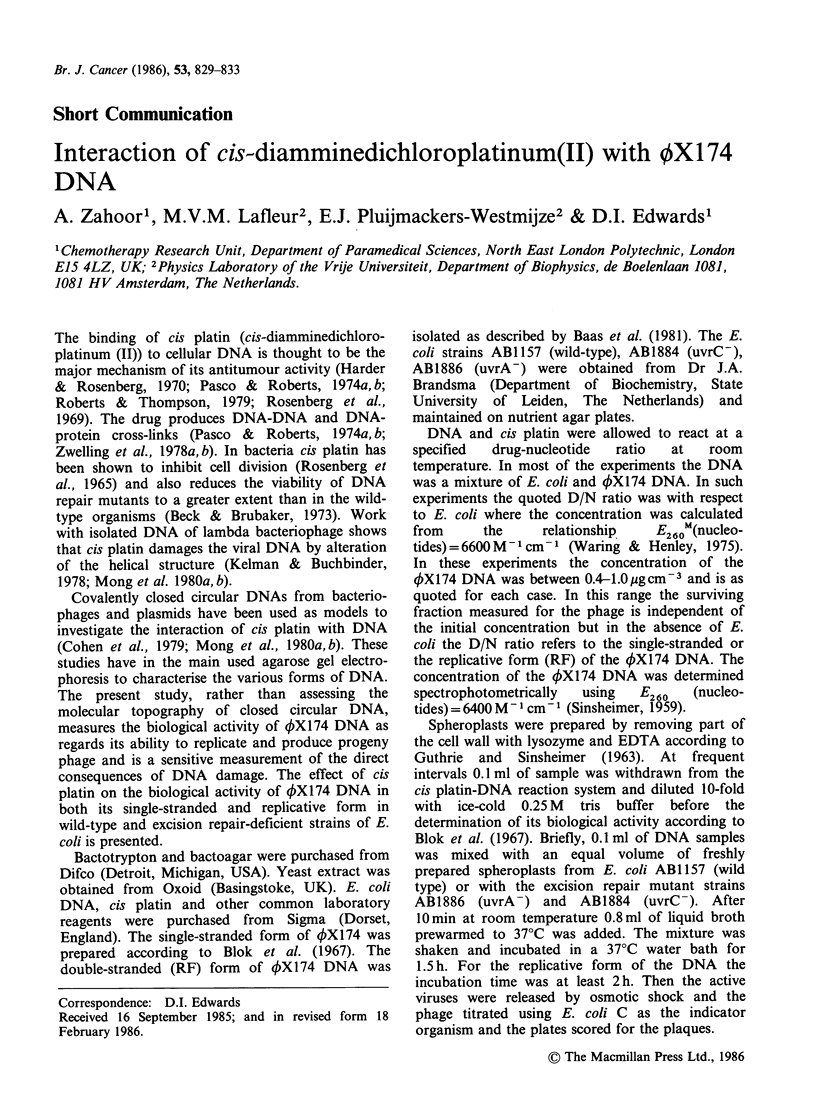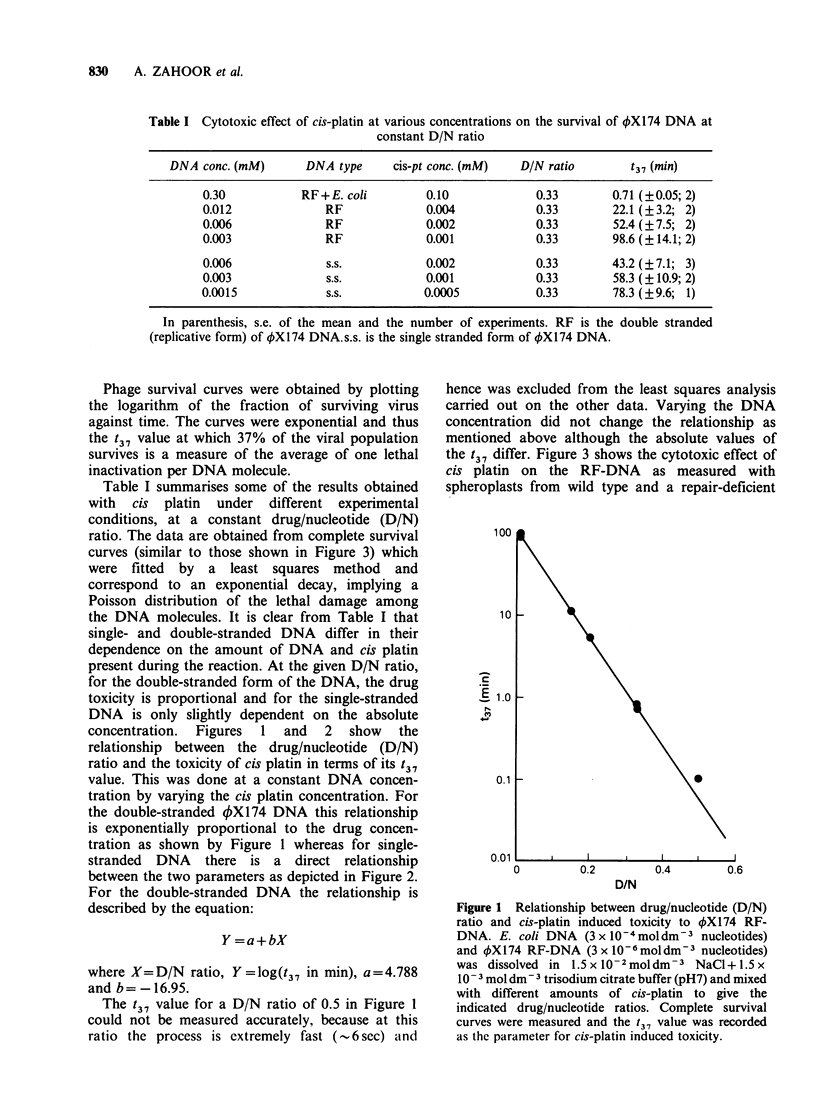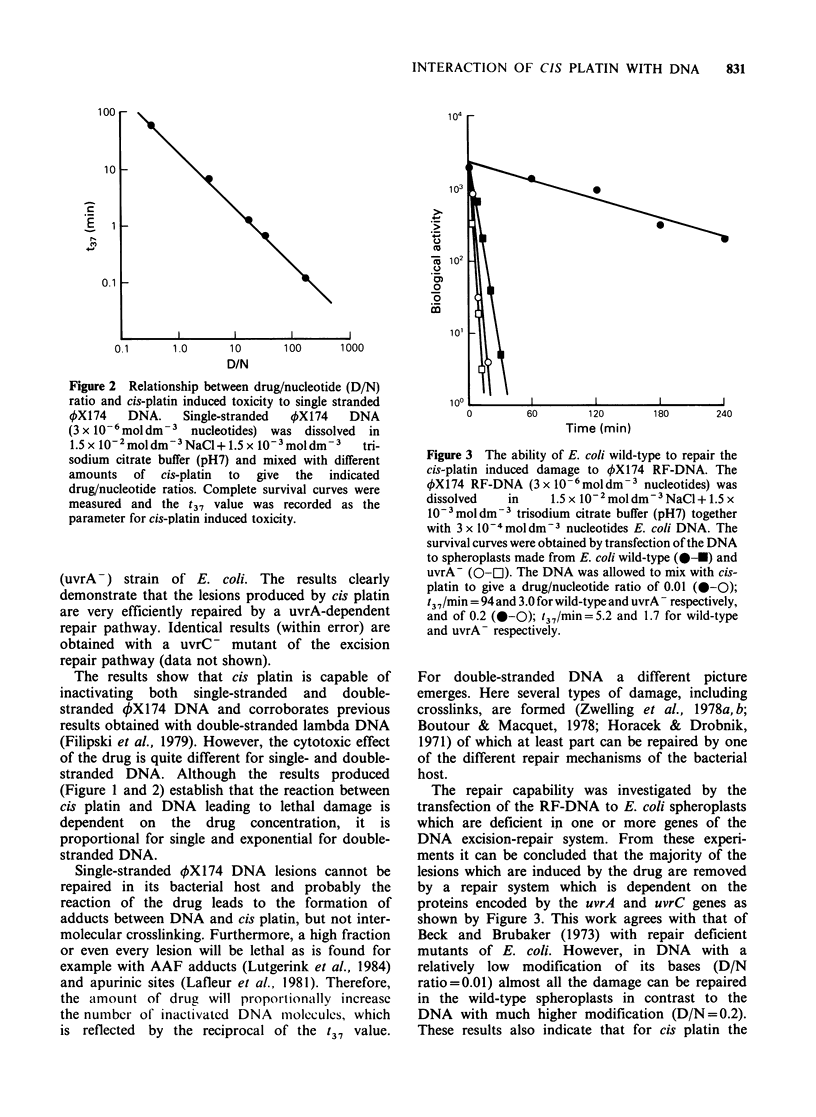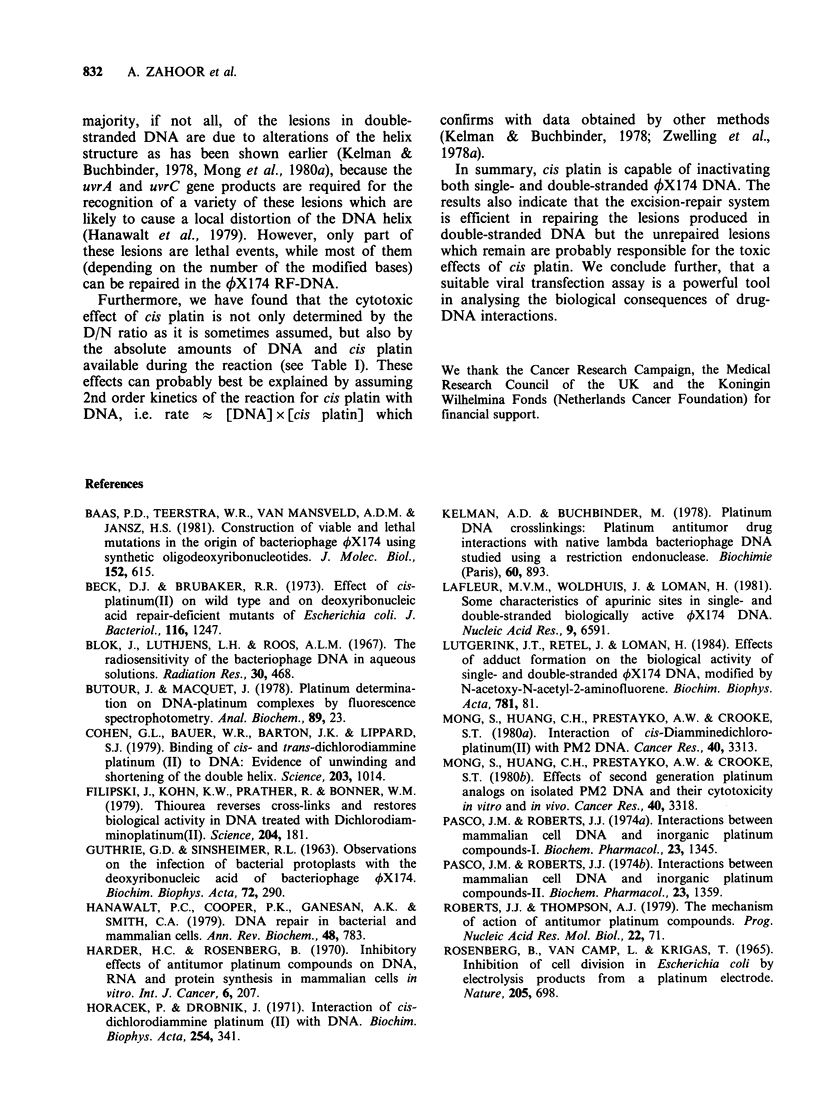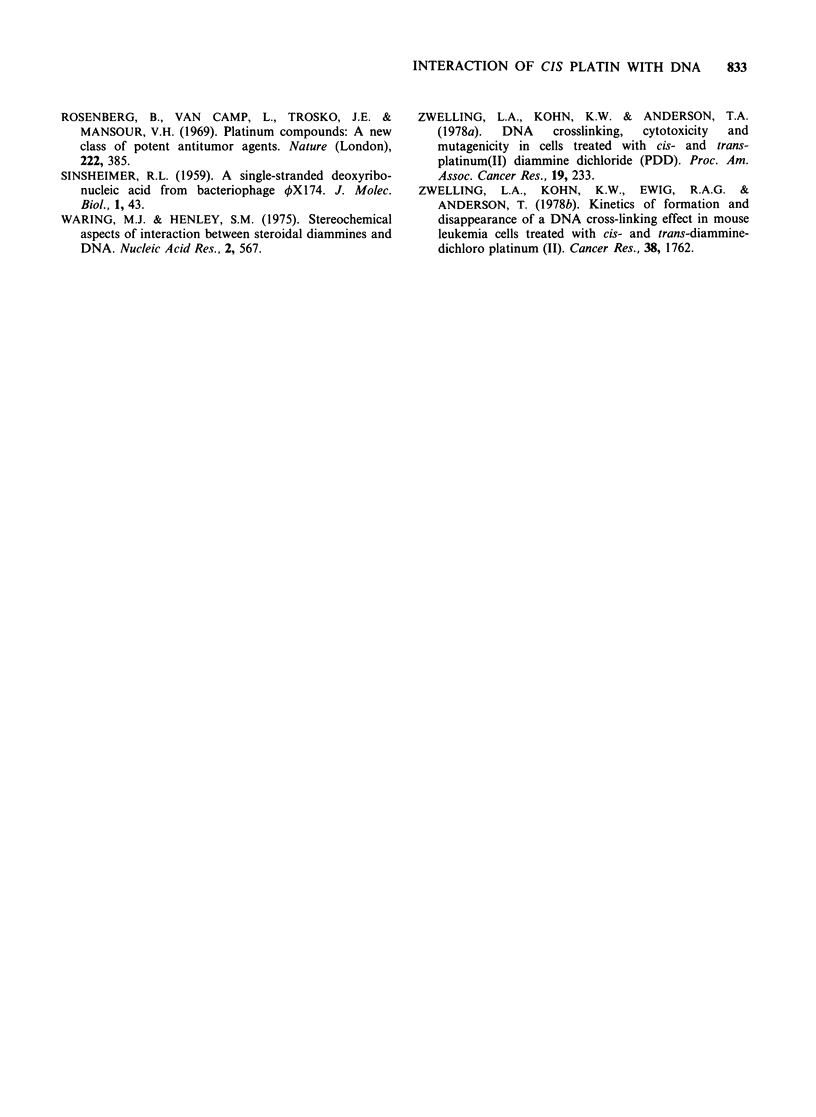# Interaction of cis-diamminedichloroplatinum(II) with phi X174 DNA.

**DOI:** 10.1038/bjc.1986.140

**Published:** 1986-06

**Authors:** A. Zahoor, M. V. Lafleur, E. J. Pluijmackers-Westmijze, D. I. Edwards


*Br. J. Cancer* (1986), **53**, 829–833

### Short Communication

### Interaction of *cis*-diamminedichloroplatinum(II) with $\phi$X174 DNA

A. Zahoor[1], M.V.M. Lafleur[2], E.J. Pluijmackers-Westmijze[2] & D.I. Edwards[1]

[1]*Chemotherapy Research Unit, Department of Paramedical Sciences, North East London Polytechnic, London E15 4LZ, UK;* [2]*Physics Laboratory of the Vrije Universiteit, Department of Biophysics, de Boelenlaan 1081, 1081 HV Amsterdam, The Netherlands.*

The binding of *cis* platin (*cis*-diamminedichloroplatinum (II)) to cellular DNA is thought to be the major mechanism of its antitumour activity (Harder & Rosenberg, 1970; Pasco & Roberts, 1974*a,b*; Roberts & Thompson, 1979; Rosenberg *et al.*, 1969). The drug produces DNA-DNA and DNA-protein cross-links (Pasco & Roberts, 1974*a,b*; Zwelling *et al.*, 1978*a,b*). In bacteria *cis* platin has been shown to inhibit cell division (Rosenberg *et al.*, 1965) and also reduces the viability of DNA repair mutants to a greater extent than in the wild-type organisms (Beck & Brubaker, 1973). Work with isolated DNA of lambda bacteriophage shows that *cis* platin damages the viral DNA by alteration of the helical structure (Kelman & Buchbinder, 1978; Mong *et al.* 1980*a,b*).

Covalently closed circular DNAs from bacteriophages and plasmids have been used as models to investigate the interaction of *cis* platin with DNA (Cohen *et al.*, 1979; Mong *et al.*, 1980*a,b*). These studies have in the main used agarose gel electrophoresis to characterise the various forms of DNA. The present study, rather than assessing the molecular topography of closed circular DNA, measures the biological activity of $\phi$X174 DNA as regards its ability to replicate and produce progeny phage and is a sensitive measurement of the direct consequences of DNA damage. The effect of *cis* platin on the biological activity of $\phi$X174 DNA in both its single-stranded and replicative form in wild-type and excision repair-deficient strains of *E. coli* is presented.

Bactotrypton and bactoagar were purchased from Difco (Detroit, Michigan, USA). Yeast extract was obtained from Oxoid (Basingstoke, UK). *E. coli* DNA, *cis* platin and other common laboratory reagents were purchased from Sigma (Dorset, England). The single-stranded form of $\phi$X174 was prepared according to Blok *et al.* (1967). The double-stranded (RF) form of $\phi$X174 DNA was isolated as described by Baas *et al.* (1981). The *E. coli* strains AB1157 (wild-type), AB1884 ($uvrC^-$), AB1886 ($uvrA^-$) were obtained from Dr J.A. Brandsma (Department of Biochemistry, State University of Leiden, The Netherlands) and maintained on nutrient agar plates.

DNA and *cis* platin were allowed to react at a specified drug-nucleotide ratio at room temperature. In most of the experiments the DNA was a mixture of *E. coli* and $\phi$X174 DNA. In such experiments the quoted D/N ratio was with respect to *E. coli* where the concentration was calculated from the relationship $E_{260}{}^{M}$(nucleotides) $= 6600\,M^{-1}\,cm^{-1}$ (Waring & Henley, 1975). In these experiments the concentration of the $\phi$X174 DNA was between $0.4–1.0\,\mu g\,cm^{-3}$ and is as quoted for each case. In this range the surviving fraction measured for the phage is independent of the initial concentration but in the absence of *E. coli* the D/N ratio refers to the single-stranded or the replicative form (RF) of the $\phi$X174 DNA. The concentration of the $\phi$X174 DNA was determined spectrophotometrically using $E_{260}$ (nucleotides) $= 6400\,M^{-1}\,cm^{-1}$ (Sinsheimer, 1959).

Spheroplasts were prepared by removing part of the cell wall with lysozyme and EDTA according to Guthrie and Sinsheimer (1963). At frequent intervals 0.1 ml of sample was withdrawn from the *cis* platin-DNA reaction system and diluted 10-fold with ice-cold 0.25 M tris buffer before the determination of its biological activity according to Blok *et al.* (1967). Briefly, 0.1 ml of DNA samples was mixed with an equal volume of freshly prepared spheroplasts from *E. coli* AB1157 (wild type) or with the excision repair mutant strains AB1886 ($uvrA^-$) and AB1884 ($uvrC^-$). After 10 min at room temperature 0.8 ml of liquid broth prewarmed to 37°C was added. The mixture was shaken and incubated in a 37°C water bath for 1.5 h. For the replicative form of the DNA the incubation time was at least 2 h. Then the active viruses were released by osmotic shock and the phage titrated using *E. coli* C as the indicator organism and the plates scored for the plaques.

Correspondence: D.I. Edwards
Received 16 September 1985; and in revised form 18 February 1986.

© The Macmillan Press Ltd., 1986

**Table I** Cytotoxic effect of *cis*-platin at various concentrations on the survival of $\phi$X174 DNA at constant D/N ratio

| *DNA conc. (mM)* | *DNA type* | cis-*pt conc. (mM)* | *D/N ratio* | $t_{37}$ *(min)* |
|---|---|---|---|---|
| 0.30 | RF + *E. coli* | 0.10 | 0.33 | 0.71 (±0.05; 2) |
| 0.012 | RF | 0.004 | 0.33 | 22.1 (±3.2; 2) |
| 0.006 | RF | 0.002 | 0.33 | 52.4 (±7.5; 2) |
| 0.003 | RF | 0.001 | 0.33 | 98.6 (±14.1; 2) |
| 0.006 | s.s. | 0.002 | 0.33 | 43.2 (±7.1; 3) |
| 0.003 | s.s. | 0.001 | 0.33 | 58.3 (±10.9; 2) |
| 0.0015 | s.s. | 0.0005 | 0.33 | 78.3 (±9.6; 1) |

In parenthesis, s.e. of the mean and the number of experiments. RF is the double stranded (replicative form) of $\phi$X174 DNA.s.s. is the single stranded form of $\phi$X174 DNA.

Phage survival curves were obtained by plotting the logarithm of the fraction of surviving virus against time. The curves were exponential and thus the $t_{37}$ value at which 37% of the viral population survives is a measure of the average of one lethal inactivation per DNA molecule.

Table I summarises some of the results obtained with *cis* platin under different experimental conditions, at a constant drug/nucleotide (D/N) ratio. The data are obtained from complete survival curves (similar to those shown in Figure 3) which were fitted by a least squares method and correspond to an exponential decay, implying a Poisson distribution of the lethal damage among the DNA molecules. It is clear from Table I that single- and double-stranded DNA differ in their dependence on the amount of DNA and *cis* platin present during the reaction. At the given D/N ratio, for the double-stranded form of the DNA, the drug toxicity is proportional and for the single-stranded DNA is only slightly dependent on the absolute concentration. Figures 1 and 2 show the relationship between the drug/nucleotide (D/N) ratio and the toxicity of *cis* platin in terms of its $t_{37}$ value. This was done at a constant DNA concentration by varying the *cis* platin concentration. For the double-stranded $\phi$X174 DNA this relationship is exponentially proportional to the drug concentration as shown by Figure 1 whereas for single-stranded DNA there is a direct relationship between the two parameters as depicted in Figure 2. For the double-stranded DNA the relationship is described by the equation:

$$Y = a + bX$$

where $X$ = D/N ratio, $Y = \log(t_{37}$ in min), $a = 4.788$ and $b = -16.95$.

The $t_{37}$ value for a D/N ratio of 0.5 in Figure 1 could not be measured accurately, because at this ratio the process is extremely fast (~6 sec) and hence was excluded from the least squares analysis carried out on the other data. Varying the DNA concentration did not change the relationship as mentioned above although the absolute values of the $t_{37}$ differ. Figure 3 shows the cytotoxic effect of *cis* platin on the RF-DNA as measured with spheroplasts from wild type and a repair-deficient

**Figure 1** Relationship between drug/nucleotide (D/N) ratio and *cis*-platin induced toxicity to $\phi$X174 RF-DNA. *E. coli* DNA ($3 \times 10^{-4}$ mol dm$^{-3}$ nucleotides) and $\phi$X174 RF-DNA ($3 \times 10^{-6}$ mol dm$^{-3}$ nucleotides) was dissolved in $1.5 \times 10^{-2}$ mol dm$^{-3}$ NaCl + $1.5 \times 10^{-3}$ mol dm$^{-3}$ trisodium citrate buffer (pH7) and mixed with different amounts of *cis*-platin to give the indicated drug/nucleotide ratios. Complete survival curves were measured and the $t_{37}$ value was recorded as the parameter for *cis*-platin induced toxicity.

**Figure 2** Relationship between drug/nucleotide (D/N) ratio and *cis*-platin induced toxicity to single stranded $\phi$X174 DNA. Single-stranded $\phi$X174 DNA ($3 \times 10^{-6}$ mol dm$^{-3}$ nucleotides) was dissolved in $1.5 \times 10^{-2}$ mol dm$^{-3}$ NaCl + $1.5 \times 10^{-3}$ mol dm$^{-3}$ trisodium citrate buffer (pH7) and mixed with different amounts of *cis*-platin to give the indicated drug/nucleotide ratios. Complete survival curves were measured and the $t_{37}$ value was recorded as the parameter for *cis*-platin induced toxicity.

**Figure 3** The ability of *E. coli* wild-type to repair the *cis*-platin induced damage to $\phi$X174 RF-DNA. The $\phi$X174 RF-DNA ($3 \times 10^{-6}$ mol dm$^{-3}$ nucleotides) was dissolved in $1.5 \times 10^{-2}$ mol dm$^{-3}$ NaCl + $1.5 \times 10^{-3}$ mol dm$^{-3}$ trisodium citrate buffer (pH7) together with $3 \times 10^{-4}$ mol dm$^{-3}$ nucleotides *E. coli* DNA. The survival curves were obtained by transfection of the DNA to spheroplasts made from *E. coli* wild-type (●–■) and uvrA$^-$ (○–□). The DNA was allowed to mix with *cis*-platin to give a drug/nucleotide ratio of 0.01 (●–○); $t_{37}$/min = 94 and 3.0 for wild-type and uvrA$^-$ respectively, and of 0.2 (●–○); $t_{37}$/min = 5.2 and 1.7 for wild-type and uvrA$^-$ respectively.

(uvrA$^-$) strain of *E. coli.* The results clearly demonstrate that the lesions produced by *cis* platin are very efficiently repaired by a uvrA-dependent repair pathway. Identical results (within error) are obtained with a uvrC$^-$ mutant of the excision repair pathway (data not shown).

The results show that *cis* platin is capable of inactivating both single-stranded and double-stranded $\phi$X174 DNA and corroborates previous results obtained with double-stranded lambda DNA (Filipski *et al.,* 1979). However, the cytotoxic effect of the drug is quite different for single- and double-stranded DNA. Although the results produced (Figure 1 and 2) establish that the reaction between *cis* platin and DNA leading to lethal damage is dependent on the drug concentration, it is proportional for single and exponential for double-stranded DNA.

Single-stranded $\phi$X174 DNA lesions cannot be repaired in its bacterial host and probably the reaction of the drug leads to the formation of adducts between DNA and *cis* platin, but not inter-molecular crosslinking. Furthermore, a high fraction or even every lesion will be lethal as is found for example with AAF adducts (Lutgerink *et al.,* 1984) and apurinic sites (Lafleur *et al.,* 1981). Therefore, the amount of drug will proportionally increase the number of inactivated DNA molecules, which is reflected by the reciprocal of the $t_{37}$ value. For double-stranded DNA a different picture emerges. Here several types of damage, including crosslinks, are formed (Zwelling *et al.,* 1978*a,b*; Boutour & Macquet, 1978; Horacek & Drobnik, 1971) of which at least part can be repaired by one of the different repair mechanisms of the bacterial host.

The repair capability was investigated by the transfection of the RF-DNA to *E. coli* spheroplasts which are deficient in one or more genes of the DNA excision-repair system. From these experiments it can be concluded that the majority of the lesions which are induced by the drug are removed by a repair system which is dependent on the proteins encoded by the *uvrA* and *uvrC* genes as shown by Figure 3. This work agrees with that of Beck and Brubaker (1973) with repair deficient mutants of *E. coli.* However, in DNA with a relatively low modification of its bases (D/N ratio = 0.01) almost all the damage can be repaired in the wild-type spheroplasts in contrast to the DNA with much higher modification (D/N = 0.2). These results also indicate that for *cis* platin the majority, if not all, of the lesions in double-stranded DNA are due to alterations of the helix structure as has been shown earlier (Kelman & Buchbinder, 1978, Mong *et al.*, 1980*a*), because the *uvrA* and *uvrC* gene products are required for the recognition of a variety of these lesions which are likely to cause a local distortion of the DNA helix (Hanawalt *et al.*, 1979). However, only part of these lesions are lethal events, while most of them (depending on the number of the modified bases) can be repaired in the $\phi$X174 RF-DNA.

Furthermore, we have found that the cytotoxic effect of *cis* platin is not only determined by the D/N ratio as it is sometimes assumed, but also by the absolute amounts of DNA and *cis* platin available during the reaction (see Table I). These effects can probably best be explained by assuming 2nd order kinetics of the reaction for *cis* platin with DNA, i.e. rate $\approx$ [DNA] $\times$ [*cis* platin] which confirms with data obtained by other methods (Kelman & Buchbinder, 1978; Zwelling *et al.*, 1978*a*).

In summary, *cis* platin is capable of inactivating both single- and double-stranded $\phi$X174 DNA. The results also indicate that the excision-repair system is efficient in repairing the lesions produced in double-stranded DNA but the unrepaired lesions which remain are probably responsible for the toxic effects of *cis* platin. We conclude further, that a suitable viral transfection assay is a powerful tool in analysing the biological consequences of drug-DNA interactions.

We thank the Cancer Research Campaign, the Medical Research Council of the UK and the Koningin Wilhelmina Fonds (Netherlands Cancer Foundation) for financial support.

### References

BAAS, P.D., TEERSTRA, W.R., VAN MANSVELD, A.D.M. & JANSZ, H.S. (1981). Construction of viable and lethal mutations in the origin of bacteriophage $\phi$X174 using synthetic oligodeoxyribonucleotides. *J. Molec. Biol.*, **152**, 615.

BECK, D.J. & BRUBAKER, R.R. (1973). Effect of *cis*-platinum(II) on wild type and on deoxyribonucleic acid repair-deficient mutants of *Escherichia coli. J. Bacteriol.*, **116**, 1247.

BLOK, J., LUTHJENS, L.H. & ROOS, A.L.M. (1967). The radiosensitivity of the bacteriophage DNA in aqueous solutions. *Radiation Res.*, **30**, 468.

BUTOUR, J. & MACQUET, J. (1978). Platinum determination on DNA-platinum complexes by fluorescence spectrophotometry. *Anal. Biochem.*, **89**, 23.

COHEN, G.L., BAUER, W.R., BARTON, J.K. & LIPPARD, S.J. (1979). Binding of *cis*- and *trans*-dichlorodiammine platinum (II) to DNA: Evidence of unwinding and shortening of the double helix. *Science*, **203**, 1014.

FILIPSKI, J., KOHN, K.W., PRATHER, R. & BONNER, W.M. (1979). Thiourea reverses cross-links and restores biological activity in DNA treated with Dichlorodiamminoplatinum(II). *Science*, **204**, 181.

GUTHRIE, G.D. & SINSHEIMER, R.L. (1963). Observations on the infection of bacterial protoplasts with the deoxyribonucleic acid of bacteriophage $\phi$X174. *Biochim. Biophys. Acta*, **72**, 290.

HANAWALT, P.C., COOPER, P.K., GANESAN, A.K. & SMITH, C.A. (1979). DNA repair in bacterial and mammalian cells. *Ann. Rev. Biochem.*, **48**, 783.

HARDER, H.C. & ROSENBERG, B. (1970). Inhibitory effects of antitumor platinum compounds on DNA, RNA and protein synthesis in mammalian cells *in vitro*. *Int. J. Cancer*, **6**, 207.

HORACEK, P. & DROBNIK, J. (1971). Interaction of *cis*-dichlorodiammine platinum (II) with DNA. *Biochim. Biophys. Acta*, **254**, 341.

KELMAN, A.D. & BUCHBINDER, M. (1978). Platinum DNA crosslinkings: Platinum antitumor drug interactions with native lambda bacteriophage DNA studied using a restriction endonuclease. *Biochimie* (Paris), **60**, 893.

LAFLEUR, M.V.M., WOLDHUIS, J. & LOMAN, H. (1981). Some characteristics of apurinic sites in single- and double-stranded biologically active $\phi$X174 DNA. *Nucleic Acid Res.*, **9**, 6591.

LUTGERINK, J.T., RETEL, J. & LOMAN, H. (1984). Effects of adduct formation on the biological activity of single- and double-stranded $\phi$X174 DNA, modified by N-acetoxy-N-acetyl-2-aminofluorene. *Biochim. Biophys. Acta*, **781**, 81.

MONG, S., HUANG, C.H., PRESTAYKO, A.W. & CROOKE, S.T. (1980*a*). Interaction of *cis*-Diamminedichloroplatinum(II) with PM2 DNA. *Cancer Res.*, **40**, 3313.

MONG, S., HUANG, C.H., PRESTAYKO, A.W. & CROOKE, S.T. (1980*b*). Effects of second generation platinum analogs on isolated PM2 DNA and their cytotoxicity *in vitro* and *in vivo*. *Cancer Res.*, **40**, 3318.

PASCO, J.M. & ROBERTS, J.J. (1974*a*). Interactions between mammalian cell DNA and inorganic platinum compounds-I. *Biochem. Pharmacol.*, **23**, 1345.

PASCO, J.M. & ROBERTS, J.J. (1974*b*). Interactions between mammalian cell DNA and inorganic platinum compounds-II. *Biochem. Pharmacol.*, **23**, 1359.

ROBERTS, J.J. & THOMPSON, A.J. (1979). The mechanism of action of antitumor platinum compounds. *Prog. Nucleic Acid Res. Mol. Biol.*, **22**, 71.

ROSENBERG, B., VAN CAMP, L. & KRIGAS, T. (1965). Inhibition of cell division in *Escherichia coli* by electrolysis products from a platinum electrode. *Nature*, **205**, 698.

ROSENBERG, B., VAN CAMP, L., TROSKO, J.E. & MANSOUR, V.H. (1969). Platinum compounds: A new class of potent antitumor agents. *Nature* (London), **222,** 385.

SINSHEIMER, R.L. (1959). A single-stranded deoxyribonucleic acid from bacteriophage $\phi$X174. *J. Molec. Biol.,* **1,** 43.

WARING, M.J. & HENLEY, S.M. (1975). Stereochemical aspects of interaction between steroidal diammines and DNA. *Nucleic Acid Res.,* **2,** 567.

ZWELLING, L.A., KOHN, K.W. & ANDERSON, T.A. (1978*a*). DNA crosslinking, cytotoxicity and mutagenicity in cells treated with *cis-* and *trans-*platinum(II) diammine dichloride (PDD). *Proc. Am. Assoc. Cancer Res.,* **19,** 233.

ZWELLING, L.A., KOHN, K.W., EWIG, R.A.G. & ANDERSON, T. (1978*b*). Kinetics of formation and disappearance of a DNA cross-linking effect in mouse leukemia cells treated with *cis-* and *trans-*diammine-dichloro platinum (II). *Cancer Res.,* **38,** 1762.